# Effect of HLA-G5 Immune Checkpoint Molecule on the Expression of ILT-2, CD27, and CD38 in Splenic B cells

**DOI:** 10.1155/2022/4829227

**Published:** 2022-05-12

**Authors:** Hana Rohn, Cordula Lang, Sabine Schramm, Falko M. Heinemann, Mirko Trilling, Anja Gäckler, Oliver Witzke, Peter A. Horn, Vera Rebmann

**Affiliations:** ^1^Department of Infectious Diseases. West German Centre of Infectious Diseases, University Hospital Essen, University Duisburg-Essen, 45147 Essen., Germany; ^2^Institute for Transfusion Medicine, University Hospital Essen, University Duisburg-Essen, 45147 Essen., Germany; ^3^Institute for Virology, University Hospital Essen, University Duisburg-Essen, 45147 Essen., Germany; ^4^Department of Nephrology, University Hospital Essen, University Duisburg-Essen, 45147 Essen., Germany

## Abstract

The human leukocyte antigen G (HLA-G) is an immune checkpoint molecule with a complex network of interactions with several inhibitory receptors. Although the effect of HLA-G on T cells and NK cells is well studied, the effect of HLA-G on B cells is still largely elusive. B cells are of particular interest in the context of the HLA-G-ILT-2 interaction because the ILT-2 receptor is constitutively expressed on most B cells, whereas it is only present on some subsets of T and NK cells. To characterize the effect of HLA-G5 molecules on B cells, we studied splenic B cells derived from cytomegalovirus (CMV) sero-positive donors after CMV stimulation with antigens in the presence and absence of soluble HLA-G5. In the presence of HLA-G5, increased expression of the ITIM-bearing Ig-like transcript (ILT-2) was observed on B cells, but its expression was not affected by stimulation with CMV antigens. Moreover, it became evident that HLA-G5 exposure resulted in a decreased expression of CD27 and CD38 and, accordingly, in lower proportions of CD19^+^CD27^+^CD38^+^ and higher proportions of CD19^+^CD27^−^CD38^−^ B cells. Taken together, our *in vitro* findings demonstrate that soluble HLA-G5 suppresses markers of B cell activation, suggesting that HLA-G5 has an impact on splenic B cell differentiation and activation. Based on these results, further investigation regarding the role of HLA-G as a prognostic factor and a potential therapeutic agent with respect to B cell function appears reasonable.

## 1. Introduction

The non-classic HLA class I molecule G (HLA-G) is a potent immunosuppressive molecule that negatively influences the effector functions of various immune cells belonging to the innate and adaptive immune system and thereby induces immune inhibition [1, 2]. Due to these immune-regulating properties, it is regarded to be an immune checkpoint molecule [3].

Several immunosuppressive mechanisms mediated by cell-associated and soluble HLA-G (sHLA-G) molecules have been described [3, 4]. HLA-G can induce either direct or indirect and either long- or short-term immune suppression. Fundamental for these immunomodulatory effects of HLA-G is the interaction with its specific inhibitory receptors, being the immunoglobulin-like transcript (ILT)-2, ILT-4, and killer inhibitory receptor (KIR) 2DL4, expressed on various immune cells [4–9]. By directly binding to these immune receptors, HLA-G and its soluble forms can exert multiple immunosuppressive functions resulting in the inhibition of immune effector proliferation, cytotoxicity, and anti-inflammatory cytokines release such as interferon, chemotaxis, and immunoglobulin production [10]. These characteristics have made HLA-G a key molecule in situations that require immune tolerance [11, 12].

Initially, the biological functions of HLA-G were recognized in the context of its function in maternal–fetal tolerance. Under physiological conditions, the expression of HLA-G is restricted to immune-privileged adult tissue [13, 14]. Over the past decades, however, HLA-G expression has also been documented in various pathological situations, such as malignancies, infections, and allograft transplantation [3, 15–21]. In such situations, increased HLA-G or sHLA-G expression allows viruses and tumors to escape from immune surveillance by inhibition of B cells, T cells, and NK cells [3, 4, 22]. Through alternative splicing, HLA-G can be either expressed as membrane-bound (HLA-G1, HLA-G2, HLA-G3, and HLA-G4) or secreted soluble (HLA-G5, HLA-G6, and HLA-G7) isoforms. The HLA-G1 molecule and its secreted soluble counterpart HLA-G5 have been the most studied. Their extracellular structure is identical to that of classical HLA class I molecules: an H chain comprising three globular domains non-covalently bound to *β*2-microglobulin (*β*2m) and a nonapeptide [23]. Unlike classic HLA-class I molecules, HLA-G exhibits only a few polymorphisms in its coding region. For HLA-G, some dimorphisms in noncoding regions have also been described. These dimorphisms affect HLA-G expression levels and thereby support immune escape mechanisms, which are often associated with adverse clinical courses [24–31].

Although the role of HLA-G in B cell malignancies has been studied before [32–38], information about the influence of HLA-G on B cells derived from immunologically healthy persons is still limited [39]. However, B cells are of particular interest in the context of interactions between HLA-G and its cognate receptor ILT-2 because ILT-2 is constitutively expressed in the B cell population, whereas it is only present in subsets of T and NK cells [8, 40]. In addition, no information is available about the effects of sHLA-G on B cells derived from secondary lymphoid organs, such as the spleen. The spleen is a key lymphoid organ for generating B cell-mediated humoral immunity. It contains distinct B cell lineages, including follicular and marginal zone (MZ) B cells. While follicular B cells recirculate and mainly participate in T cell-dependent immune responses, MZ B cells occupy strategic locations for monitoring the presence of blood-borne and mucosa-associated pathogens [41]. Almost all MZ B cells in the adult spleen express CD27, a molecule known to be a costimulatory receptor belonging to the tumor necrosis factor (TNF) receptor superfamily involved in T and B cell interactions [42, 43]. CD27 has been identified as a marker of memory B cells [44]. In addition to CD27, CD38 is another important surface receptor involved in the regulation of B cell activation and maturation in the spleen [45]. It is highly and continuously expressed in plasma cells, thus making it an ideal target for multiple myeloma treatment with monoclonal antibodies against CD38 [46].

To gain further insight into the functional implication of sHLA-G on splenic CD27^+/-^CD38^+/-^ B cell subsets, we primed spleen-derived mononuclear cells (MC) from cytomegalovirus (CMV) positive immunologically healthy cadaveric organ donors with HLA-G5 with or without CMV antigen stimulation.

## 2. Materials and Methods

### 2.1. Subject and Samples

Spleen samples collected at the Institute of Transfusion Medicine, University Hospital Essen, from anonymous, immunologically healthy, cadaveric organ donors, and splenic mononuclear cells (MCs) were isolated. All samples were collected from leftover material. The study was approved by the local ethics committee (approval no. 17-7888-BO) and was performed in accordance with the ethical standards noted in the 1964 Declaration of Helsinki and its later amendments or comparable ethics standards. All 5 splenic MC samples were collected from CMV IgG positive adults.

To isolate splenic MCs, we flushed a piece of spleen several times with body warmth RPMI-1640 medium (Gibco, Karlsruhe, Germany) and 1% heparin. The splenic MCs of the respective cell suspensions were separated by Ficoll density gradient centrifugation. Cell counts were determined with a Sysmex automated hematology analyzer (Sysmex Corporation, Norderstedt, Germany) according to the manufacturer's protocol. For vitality control, we also determined the cell count with a BLAUBRANDR® counting chamber. In addition to lymphocytes, the MCs of the spleen samples also consist of up to approximately 20% monocytes. Because the monocytes could interfere with additional experiments because of phagocytosis, they were removed by adhering to the plastic walls of the cell culture plates, as previously described [47]. Splenic MCs were cryopreserved with custodiol/10% DMSO and carefully thawed for stimulation experiments.

### 2.2. Stimulation with CMV and HLA-G5 Molecules

For each condition, we plated 2.5 × 106 splenic MCs in 96-well U-bottom plates in RPMI-1640 media, supplemented with 1% penicillin–streptomycin–glutamine (Gibco, Karlsruhe, Germany), 1% FCS Gold (PAA, Cölbe, Germany), 0.1 mM nonessential amino acids, 1 mM pyruvate, 200 U/ml IL-2, 50 ng/ml IL-10, 40 *μ*g/ml apo-transferrin, and 5% CO2 at a density of 0.5 ×106 cells per well in a total volume of 200 *μ*L at 37 °C. Splenic MCs were stimulated overnight (24 h) with CMV, HLA-G5 molecules, or both antigens simultaneously or were left untreated as controls.

Splenic MCs were stimulated with a complex mixture of CMV antigens (derived from the HCMV strain AD169) at a final concentration of 16.7 *μ*g/ml (Microbix, Ontario, Canada) and in house-produced antigens generated from MRC-5 cells infected with HCMV-TB40 strain [48, 49] at a final protein concentration of 33 *μ*g/ml. Biologically active HLA-G5 [50] was used at a final concentration of 100 ng/ml.

### 2.3. Flow Cytometry Analysis

To study ILT-2 surface expression on splenic B cells, we stained the cell suspension with anti-human CD19 (APC, clone SJ25C1, eBioscience, Frankfurt Germany) and anti-human ILT-2 (PE, clone GHI/7, BD Bioscience, Heidelberg, Germany). For surface staining of CD19, CD27, and CD38 on splenic B cells, the following monoclonal antibodies were applied: anti-human CD19 (PE, clone HIB19), anti-human CD27 (FITC, clone O323), and anti-human CD38 (APC, clone HIT2). All antibodies were provided by eBioscience. Isotype-matched antibodies served as negative controls (BD Bioscience). Stained samples were assessed in a FACSCalibur analysis, data acquisition was performed with CellQuest software (BD), and data were analyzed by Kaluza Analysis Software Version 2.1 (Beckman Coulter, Brea, California USA). The expression of antigens is given as the index of mean fluorescence intensity (MFI) was calculated as follows: (MFI sample–MFI IgG control)/MFI IgG control.

### 2.4. Statistical Analysis

Statistical analysis was performed with GraphPad Prism V8.4.3 (GraphPad Software, San Diego, CA, USA). The expression of cell marker (MFI) is provided as mean ± standard error of the mean. Continuous variables were compared with two-tailed paired *t*-tests after testing for Gaussian distribution. Statistical significance was set at the level of *P* *values* ≤ 0.05.

## 3. Results

### 3.1. HLA-G5 Modulates the ILT-2 Expression of Splenic B Cells

The immunomodulatory effect of HLA-G5 is typically mediated by its interaction with ILT-2 receptor. In addition, ILT-2 is the only known HLA-G-specific receptor expressed by CD19^+^ B cells [8, 40]. To study the effect of HLA-G5 on ILT-2 receptor expression in CD19^+^ cells derived from secondary lymphoid organs, we primed splenic MCs derived from CMV positive donors (*n* = 5) with HLA-G5 overnight in the presence or absence of CMV-derived antigens. The following conditions were assessed: splenic MCs were cultured either (i) untreated as negative controls or (ii) cultured with HLA-G5 alone, (iii) cultured with CMV antigens alone or (iv) cultured with both CMV antigens, and HLA-G5 simultaneously (iv).

**Figure 1:**
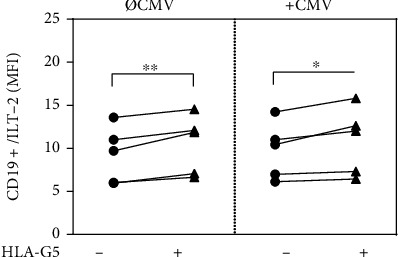
Incubation with Human leukocyte antigen G5 (HLA-G5) significantly increases the surface expression of ITIM-bearing Ig-like transcript (ILT-2) in CD19+ splenic cells. Differences in ILT-2 expression on splenic CD19+ cells derived from cytomegalovirus (CMV)-seropositive individuals (*n* = 5) after overnight cultivation in the presence or absence of HLA-G5 and CMV antigens. The expression of ILT-2 was analyzed by flow cytometry. The ILT-2 expression is given as the mean fluorescence intensity index (MFI). Statistical significance (*P* < 0.05) was determined by two-tailed paired *t*-test. ∗*P* < 0.05, ∗∗*P* < 0.01.

A flow cytometric analysis showed that ILT-2 receptor expression [mean fluorescence intensity (MFI) ± standard error of mean (SEM)] is not significantly different between CD19^+^ cells either stimulated (9.76 ± 1.47) or not stimulated (9.26 ± 1.47; *P* = ns) with CMV. Incubation with HLA-G5, however, significantly increased ILT-2 expression in splenic B cells (Figure 1). Of note, this increase in ILT-2 receptor expression mediated by HLA-G5 occurs independently of stimulation with CMV-derived antigens (with stimulation, *P* = 0.0096; without stimulation, *P* = 0.04).

### 3.2. HLA-G5 Modulates CD27 Expression in CMV-Stimulated and Non-stimulated Splenic B Cells

**Figure 2:**
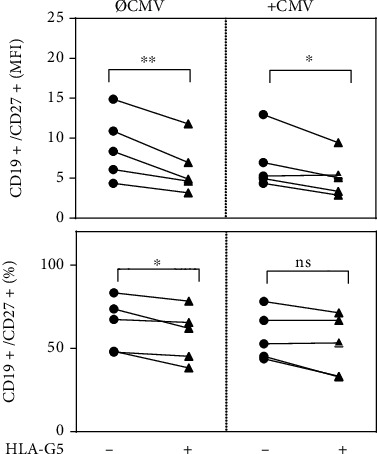
Human leukocyte antigen G5 (HLA-G5) significantly decreases the surface expression of CD27 in CD19+ splenic cells and reduces the proportion of CD19 + CD27+ cells in the total population of CD19+ cells. Differences in CD27 expression in splenic CD19+ cells derived from cytomegalovirus (CMV)-seropositive individuals (*n* = 5) after overnight cultivation with or without HLA-G5 and CMV antigens. CD27 expression was analyzed by flow cytometry and is given as mean fluorescence intensity index (MFI) and the proportion of CD19 + CD27+ cells in the total population of CD19+ cells (%). Statistical significance (*P* < 0.05) was determined by two-tailed paired *t*-test. ∗*P* < 0.05, ∗∗*P* < 0.01.

Next, we elucidated the effect of HLA-G5 on the costimulatory receptor CD27, which is involved in effector and memory differentiation of B cells. As shown in Figure 2, compared to control cells, CD19^+^ splenic B cells primed overnight with HLA-G5 exhibited significantly lower CD27 expression whether they were stimulated with CMV (*P* = 0.04) or not (*P* = 0.009). In addition to modulating CD27 expression, HLA-G5 treatment resulted in lower proportions of CD19^+^CD27^+^ cells (*P* = 0.04). Stimulation with CMV antigen alone exerted a significant, albeit marginal, effect on the CD27 expression in CD19^+^ cells (MFI ± SEM: 8.93 ± 1.85 vs. 6.91 ± 1.57; *P* = 0.04). These findings show that HLA-G5 mediates an alteration in CD27 expression and in subset frequencies of splenic B cells.

### 3.3. HLA-G5 Modulates the Expression of CD38 Exclusively in CMV-Stimulated Splenic B Cells

**Figure 3:**
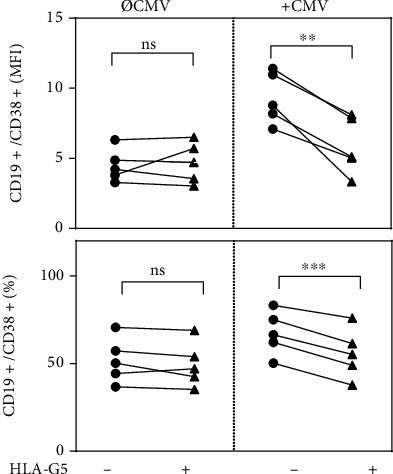
Human leukocyte antigen G5 (HLA-G5) significantly decreases the surface expression of CD38 in CD19^+^ splenic cells and decreases the proportion of CD19^+^CD38^+^ cells in the total population of CD19^+^ cells after cytomegalovirus (CMV) antigen stimulation. The expression of CD38 is given as mean fluorescence intensity index (MFI) and the proportion of CD19 + CD38+ cells in the total population of CD19^+^ cells (%). Statistical significance (*P* < 0.05) was determined by two-tailed paired *t*-tests, ns: not significant; ∗∗*P* < 0.01; ∗∗∗*P* < 0.001.

Human CD38 is a multifunctional surface molecule which serves as a marker for cellular activation and proliferation [51]. Because of the immunosuppressive properties of HLA-G5, we analyzed CD38 expression in splenic B cells either primed with HLA-G5 or not primed and treated with CMV antigens or untreated. The analysis showed that the CD38 expression (MFI ± SEM) in CD19^+^ cells increased significantly (*P* < 0.00001) after stimulation with CMV antigens (9.28 ± 0.82) compared to non-stimulated cells (4.50 ± 0.52). Remarkably, although overnight priming with HLA-G5 alone (i.e., in the absence of CMV antigens) had no effect on CD38 expression, HLA-G counteracted the effect induced by CMV antigen stimulation. As a result, CD38 expression was significantly decreased (*P* = 0.004) as measured by MFI in CD19^+^ cells, as well as decreased in the proportion of CD19^+^CD38^+^ cells in the total population of CD19^+^ cells (*P* = 0.0006) (Figure 3).

### 3.4. HLA-G5 Modulates the Proportion of CD27^+^38^+^ Cells in a Population of Splenic B Cells

**Figure 4:**
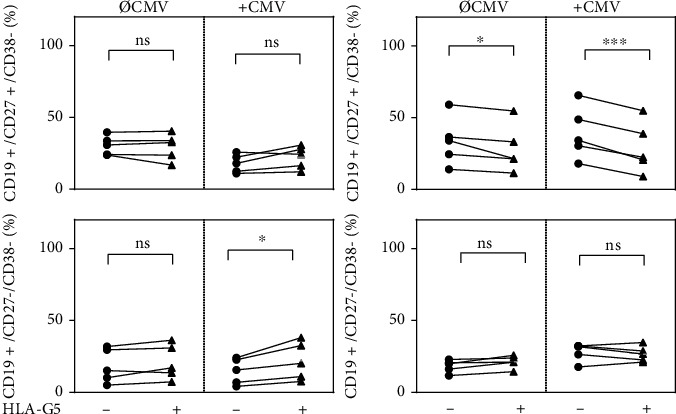
Human leukocyte antigen G5 (HLA-G5) significantly decreases the proportion of CD27^+^CD38^+^ in the total population of splenic B cells and increases the proportion of CD27^−^CD38^−^ B cells. CD27 and CD38 were analyzed by flow cytometry. . Statistical significance (*P* < 0.05) was determined by two-tailed *t-*test. ns: not significant; ∗*P* < 0.05; ∗∗∗*P* < 0.001.

Because CD27 and CD38 are important surface markers of B cell differentiation, we focused on the combined results of CD27 and CD38 modulation induced by HLA-G5 in splenic B cells stimulated or not stimulated with CMV antigens. A statistically significant reduction in the proportion of CD27 + C38+ splenic B cells was observed in the presence of HLA-G5, usually considered plasmablasts (*P* = 0.046) [52]. This finding was even more pronounced (*P* = 0.0004) under the influence of CMV antigen stimulation (Figure 4). In parallel, HLA-G priming significant increased proportion of CD27^−^CD38^−^ splenic B cells only in conjunction with CMV stimulation.

Taken together, the combined results demonstrate that HLA-G5 affects the proportion of B cell subpopulations because it reduces the number of activated CD27^+^CD38^+^ B cells and increases the expression of the inhibitory molecule ILT-2. These effects may promote B cell inhibition.

## 4. Discussion

HLA-G is an important immune modulator belonging to the group of non-classical HLA class Ib molecules. Because of its complex interaction network with specific inhibitory receptors on immune effector cells, it is considered a relevant immune checkpoint molecule. Over the past decades, aberrant HLA-G expression has been reported in various pathological situations, such as malignancy, infections, and transplantation [1, 3]. Multiple studies have linked increased HLA-G expression to disease outcomes [2, 4, 28]. Overall, the presence of HLA-G molecules in both membrane-bound and soluble isoforms has been associated with tolerogenic functions on both innate and adaptive cellular responses. The effects of HLA-G on T cells and NK cells have been extensively studied under the aforementioned conditions [4]. In contrast, the effect of HLA-G on B cell function remained largely elusive. B cells execute a multitude of effector functions, including the secretion of antibodies, presentation of antigens, production of cytokines, and formation of immunologic memory [53]. Regarding the clinical context, the presence of sHLAG in patient serum is associated with the progression of B cell malignancies [32]. Interestingly, the group of Terasaki associated the presence of sHLA-G in patient sera with a lower incidence of HLA-IgG antibodies after transplantation, thus potentially reflecting impaired B cell activity [54]. In line with this finding, circulating sHLA-G levels are lower after heart transplantation among patients with biopsy proven humoral rejection than among patients without evidence of rejection [55]. These two studies provide evidence that sHLA-G may be involved in modulating B cell function *in vivo*, in accordance with *in vitro* experiments [39].

In the present study, we investigated the effect of HLA-G5 on B cells derived from the spleen *in vitro*. Overall, our work found that HLA-G5 (i) increases the expression of the inhibitory molecule ILT-2 on splenic B cells and (ii) decreases the proportion of activated CD19^+^CD27^+^CD38^+^ while increasing the proportion of CD19^+^CD27^−^CD38^−^ cells, which are considered plasmablasts or naïve B cells.

Immunosuppressive functions of HLA-G are mediated by inhibitory ILT-2 and ILT-4 signaling. The importance of this interaction has been well described, rendering the HLA-G/ILT-2 ligand-receptor axis one of the most promising new therapeutic targets for immune checkpoint inhibitors [56, 57]. ILT-2 is expressed in various intensities by most monocytes, dendritic cells, and B cells as well as by some NK and T cell subsets. Of note, ILT-2 on CD19+ B cells is the sole known HLA-G-specific receptor to be expressed [6, 40]. Therefore, the question of reciprocal ligand-receptor interference is of high biological relevance for understanding the effect of HLA-G on B cell function.

The upregulation of inhibitory receptors in immune cells may increase their activation thresholds; thus, this upregulation is an important mechanism of immune defense. Previous studies have shown that HLA-G1 and its soluble isoform HLA-G5 can induce transcriptional and phenotypical changes in immune cells as described by the upregulation of ILT-2, ILT-3, ILT-4, and KIR2DL4 on antigen-presenting cells, NK cells, and T cells [58]. This upregulation does not appear to require antigen stimulation. Our study expanded this list of affected immune cells by adding splenic B cells. Thus, the findings of our study are in line with those of an *in vitro* study that demonstrated that HLA-G aggregates on-to nanoparticles and suppresses B cell function through ILT-2 receptor interactions [39]. This inhibition mediated by HLA-G targets the proliferation of B cells, as well as the differentiation of B cells into antibody-secreting cells [39]. However, in contrast to our findings, the earlier study examined not only the effect of HLA-G on B cells in peripheral blood but also the effect of HLA-G aggregated onto nanoparticles as primers for condensing the local concentration [39]. This difference may have altered the concentration of HLA-G in the microenvironment of B cells *in vitro*. In the present work, we chose a more physiologic approach: We used free biologically active HLA-G5 molecules associated with *β*2-microglobulin. These molecules have previously been shown to functionally interact with the ILT-2 receptor [50].

The HLA-G5 isoform has been shown to upregulate the inhibitory receptors on the cell surface more potently than does the membrane-bound HLA-G1, although these two molecules differ only slightly in their structure [58]. Thus, we hypothesized that HLA-G acts as a cell-to-cell signaling molecule. HLA-G-induced upregulation of inhibitory receptor expression may initiate an immune response because it occurs in the absence of concurrent antigen-dependent activation. For the study reported here, we chose CMV-derived antigens as a specific B cell stimulus. CMV, a member of the *Betaherpesvirinae* subfamily, establishes lifelong persistence after primary infection and has a high sero-prevalence [59]. In immunocompetent adults, CMV infection is usually subclinical, but it causes severe morbidity and mortality in patients with acquired immunodeficiency, such as transplant recipients [60]. CMV has evolved a number of mechanisms for evading the immune system, including targeting of the HLA-G-ILT-2 axis to immune effector cells [61].

After nonspecific stimulation with pokeweed mitogen and specific stimulation with tetanus toxin, the interaction of HLA-G and ILT-2 leads to reduced antibody production [39]. Our study provides the first evidence of a direct link between the presence of HLA-G5 and altered expression of the activation markers CD27 and CD38 and the proportion of B cell subpopulations.

Co-cultivation with HLA-G5 and CMV antigens resulted in a significant decrease in the number of CD19^+^CD27^+^CD38^+^ B cells and an increase of CD19^+^CD27^−^CD38^−^ B cells. CD27 is a member of the tumor necrosis factor receptor superfamily that regulates lymphocyte function; it has been identified as a hallmark of memory B cells [44]. After CD27 stimulation, enhanced effector and memory differentiation of immune effector cells occurs [62, 63]. In B cells, CD27 plays a key role in T dependent B cell responses and is responsible for plasma cell differentiation [64]. Thus, the CD27^−^ B cell compartment comprises mainly naïve B cells, and CD27^+^ B cells comprise memory or plasma cells [65]. CD38 is an additional important surface receptor that plays an essential role in the regulation of B cell maturation in the spleen [45]. Because human CD38 is highly expressed both on germinal center B cells and plasma cells, it has been extensively used as a marker of B cell activation [66]. In this sense, many studies support the importance of CD38 in maintaining proinflammatory profiles in innate immune cells [67].

Consequently, all of these effects of HLA-G5 on B cells converge to activate inhibitors or inhibit activators of cell survival and proliferation. Altogether, our *in vitro* data show that HLA-G plays a crucial role in regulating splenic B cell fate decisions.

## Data Availability

The raw data supporting the conclusions of this article will be made available by the authors upon request.
